# Assessing the modifiable and non-modifiable risk factors associated with multimorbidity in reproductive aged women in India

**DOI:** 10.1186/s12889-024-18186-6

**Published:** 2024-03-04

**Authors:** Priya Das, Subhadeep Saha, Tanu Das, Partha Das, Tamal Basu Roy

**Affiliations:** 1 Department of Geography, University of Gour Banga, 732101 Malda, West Bengal India; 2 Department of Geography, Raiganj University, 733134 Uttar Dinajpur, West Bengal India

**Keywords:** Multimorbidity, Modifiable and non-modifiable risk factors, Women, Reproductive age group, India

## Abstract

**Background:**

Reproductive span is the foundation of every woman’s health in later life. India is currently facing a growing burden of multiple morbidities among the women in their reproductive age group which may further increase over the coming decades. The purpose of the present study aimed to identify different modifiable and non-modifiable risk factors affecting multimorbidity among the women in reproductive age group in Indian context.

**Methods:**

Secondary data were obtained from the Demography and Health Survey (DHS), conducted in India during 2019–2021. A total of 671,967 women aged 15–49 years were selected for this present study. Descriptive, association studies and multinominal logistic regression analyses were performed to accomplish the objectives.

**Results:**

Currently, 6.3% of total study participant’s reproductive age group women suffered from multimorbidity in India. Never consuming protein, fruits, vegetables and milk increase the chances of developing multimorbidity. Consumption of fried foods, aerated drinks and addiction towards tobacco and alcohol also has a greater influence on the prevalence of multimorbidity. The prevalence of multimorbidity is sharply increased with increasing age and Body Mass Index (BMI). Regionally, the prevalence of multimorbidity was found more among the women hailed from eastern and north-eastern India.

**Conclusion:**

To reduce the risk of developing multimorbidity, targeted interventions are needed in the form of educating every woman concerning the importance of having minimum health-related knowledge, maintaining healthy lifestyle, weight management and having proper and balanced diet.

## Introduction

Currently, multimorbidity has been emerged as a serious public health concern and poses an utmost challenge to healthcare systems in all over the world. Multimorbidity, also known as multiple long-term conditions (MLTC) is defined as the coexistence of two or more chronic illnesses in an individual [1]. Nowadays through the improvements in public health and access to quality health care, people are living longer but most frequently with the problems of multimorbidity. Globally, the burden of multimorbidity is increasing day by day, accounting for 71% (41 million out of 57 million deaths) of all deaths [2].

Multimorbidity is generally associated with a greater possibility of adverse effects such as impoverished quality of life [3] & [4], greater disability and declining physical functioning [5], higher chances of mortality [6, 7] & [8] and greater financial burden on patient’s family [9] & [10].Worldwide, the prevalence of multimorbidity was around 37% [11]. Diabetes, hypertension, asthma, thyroid, heart diseases, kidney diseases, cancer etc. are some of the major non-communicable diseases considering as multimorbidity condition among people [12] & [13].

Previous studies conducted in different countries evidenced that urban population are more vulnerable to getting affected by different non-communicable diseases (NCDs) such as overweight and obesity, diabetes, hypertension, cholesterol, cardiovascular diseases and increased urbanization is also associated with the higher prevalence of those diseases [14, 15] & [16]. Urbanization is one of the most important socio-environmental factors, having a strong relationship with changing life styles and altering behavioral factors such as unhealthy diet, low physical activity, tobacco use, alcohol intake and consequently getting more affected by different NCDs [17] & [18]. Generally workers in all sectors are also at risks of NCDs. This can be prevented by improving working condition, working environment and also through workplace health promotion programmes. Workplace is where people spend maximum time in a day. Different hazardous substances including dusts, chemicals and fumes in workplace may lead to different NCDs such as respiratory disease, cardiovascular disease, cancers and diabetes etc. [19].

The risk factors of multimorbidity can be separated into two ways; modifiable factors and non-modifiable factors. Modifiable risk factors of multimorbidity are those which can be modified or controlled if one take definite measures whatever by altering their unhealthy lifestyle or others whereas non-modifiable factors includes those where modification or changes is not possible. The most commonly identified non- modifiable factors are age, sex, religion, caste, family history and genetics [20] whereas modifiable factors include different lifestyle factors. Lifestyle risk factors which one can alter by avoiding unhealthy diet, physical inactivity, exposure to tobacco smoking, excessive consumption of alcohol, sleeping irregularities etc. [21, 22] & [23]. Apart from, Education, Working status, marital status, household’s wealth quintile, residential place, religion, caste etc. are some of the important socio-economic and demographic factors also influencing multimorbidity [24] & [25]. Numerous previous studies indicated that multimorbidity is more frequent among people from higher socio-economic background [26] & [27] but contradiction was found in some other studies where multimorbidity is more widespread among those individuals belonging from poor socio-economic background [28] & [29].

Different national and state level studies conducted in India revealed that Indian women suffered more by multimorbidity than Indian men [30] & [31]. In developing countries like India, gender inequality is till now a very common deep-rooted issue [32]. Owing to this prevalent societal context, women usually face discrimination in several aspects including social, cultural, political, economical, work participation and educational settings too [33] & [34]. Even, they are deprived from basic health care system and thereby most frequently suffer with different life-threatening critical health problems [35]. From childhood to adulthood and so far, women experience inequalities in terms of food and nutrition intake, proper immunization and sleeping practices which make them vulnerable to poor physical and mental health outcomes across their entire life cycle [36].

The reproductive span that is 15–49 years is recognized as a crucial time period of every woman’s life. Women’s half of life span is elapsed through their reproductive years. Multimorbidity burden among women in reproductive age group may have critical health issues on women and child health in the early as well as later years of life. Previously enormous literature highlighted about the multimorbidity burden among older adults for different country context but there are relatively few studies focusing on identifiable risk factors dividing as modifiable and non-modifiable risk factors of multimorbidity among women in reproductive age group (15–49 years) in India. Even, whatever studies done so far with the women of reproductive ages, mainly concentrated on sexual and reproductive health aspects. Not only this, still Indian Government did not opt any national programme incorporating management of chronic illnesses during the reproductive span. It is becoming necessary to unfold this domain holistically keeping the reproductive age-grouped women at the centre. Therefore, our present study aims to determine the risk factors of multimorbidity among women in reproductive age group (15–49 years) in India.

## Study design

### Data source

The data for present study was derived from the fifth round of the National Family Health Survey (NFHS-4 &5), conducted in India during 2019-21 under the stewardship of the Ministry of Health & Family Welfare (MoHFW) and implementation was done by the International Institute of Population Sciences (IIPS), Mumbai. NFHS-5 is a source of population-based, large-scale nationally representative cross-sectional sample survey data. It collects information from 707 districts including in 29 states and 7 union territories. The survey was completed by gathering information from 636,699 households with a response rate of 98%, consisting of 724,115 women interviewed from 15 to 49 years with a response rate of 97% and 101,839 men interviewed from 15 to 54 years with a response rate of 92%. A total of four survey questionnaires-household questionnaire, woman’s questionnaire, man’s questionnaire and biomarker questionnaire were utilized in 19 local languages by Computer Assisted Personal Interviewing (CAPI). One can access this dataset freely through requesting from the online repository of the Demographic and Health Survey (DHS) (https://dhsprogram.com/data/).

### Study participants

NFHS-5 provides information about 724,115 women in reproductive age group (15–49 years). At first for the cleansing of data we’ve removed all the missing values and unnecessary responses (Don’t know) from each selected variables where it had been found that ‘Don’t know’ responses for 52,148 women. After excluding those women, all women in reproductive age group who participated in the NFHS-5 were enrolled for this present study. So, after justifying all inclusion and exclusion criterion, the final study participants for this study were considered as 671,967 women.

### Variable description

#### Outcome variable

Women multimorbidity (15–49 years) was considered as the main outcome of interest for this current study. In this regard the data coding for outcome variable was created considering the following questions-

(i) Do you currently have diabetes? (ii) Do you currently have hypertension? (iii) Do you currently have Asthma? (iv) Do you currently have Thyroid? (v) Do you currently have heart diseases? (vi) Do you currently have kidney diseases? (vii) Do you currently have cancer? Responses for all the questions were in the form of no/yes. Women’s multimorbidity was grouped into three categories (0 = no morbidity, 1 = single morbidity and 2 = multimorbidity). The women who had no disease were considered as they had no morbidity, women having only one disease were taken in the group of ‘single morbidity’ and with this, those women who had two or more of the above mentioned diseases were considered as they had multimorbidity.

#### Explanatory variables

The explanatory variables selected for present study were divided into 2 broad categories i.e. (i) Modifiable Factors and (ii) Non-modifiable factors.

##### Modifiable factors

Protein, fruits, vegetable and milk consumption, consumption of fried foods and aerated drinks, smoking tobacco, chewing tobacco, consuming alcohol and Body Mass Index (BMI) are taken as important principal modifiable factors determining women’s multimorbidity within the age group of 15–49 years. The responses of consumption of protein, fruits, vegetables and milk were in the form of daily, weekly, occasionally and never and in this study, responses were categorized into three as daily (1), weekly/occasionally (2) and never (3). If the respondents reported that they ate protein, fruits, vegetables and milk once in a week or occasionally, were taken into one single group as both the two responses were very close to each other. Consumption of fried items and aerated drinks were dichotomized into yes/no (1/0) category. Never and occasional consuming of fried items was kept in one group and was coded as ‘no’ (0) and daily and weekly consumption were kept in another group ‘yes’ (1). The variable, smoking tobacco was not found directly in NFHS-5 dataset. We have generated it by computing through several questions. Generally, bidis, cigarettes, hookah and cigar or cheroots are tobacco products consumed by smokers in India. That’s why for making this variable, we utilized the questions- (a) Do you currently smoke bidis? (b) Do you currently smoke cigarettes? (c) Do you currently smoke hookah? (d) Do you currently smoke cigar or cheroots? If the women smoked any one of the above tobacco products, were considered as smoking tobacco user (1) and otherwise not (0). Similarly, the chewing tobacco variable was also not found directly in dataset. We had to construct it by asking respondents if they consumed (a) gutkha/pan masala, (b) pan and (c) khaini. The respondents who chewed gutkha or pan or khaini were taken as chewing tobacco user. We got the variable alcohol consumption by asking the question “Do you drink alcohol?” All variables regarding lifestyle characteristics were coded as yes/no question (0/1).Women’s Body Mass Index (BMI) was also identified as one of the most important modifiable factors influencing multimorbidity. Following WHO’s criteria of classifying BMI, we classified it into four distinct categories: less than 18.5- underweight (coded as 1), 18.5 to 24.9- normal (2), 25 to 29.9- overweight (3) and 30 and higher- obese (4).

##### Non-modifiable factors

Women’s age was categorized into 3 groups such as 15–24 years (1), 25–34 years (2) and 35–49 years (3). Besides, religion (Hindu-1, Muslim-2, Christian-3 and others-4), caste/tribe (Scheduled caste-1, Scheduled Tribe-2 and Others-3) and geographical region (categorized into north-1, central-2, east-3, north-east-4, west-5 and south zones-6) were considered as most responsible non-modifiable factors affecting women’s multimorbidity. Women apart from Scheduled Caste (SC) and Scheduled Tribe (ST) categories were considered into the categories of others.

##### Covariates

Educational status of women was grouped into no education (1), primary (2), secondary (3) and higher education (4). We coded marital status of women as unmarried (1), married (2) and others (3). Here, all the widowed, divorced and separated women were kept in others group. To visualize the prevalence of women multimorbidity was greater among the mass media exposed women or not, media exposure was divided into exposed and not exposed groups. This variable was created by using following three questions, that were - (a) Do you read newspaper or magazine? (b) Do you listen to radio? (c) Do you watch television? If the respondent agreed with two amongst the three then their responses were coded as exposed (1) otherwise not exposed (2). Women’s parity was grouped into 3 classes: 0 (1), 1 (2) and 2 or more (3). ‘0’ means no parity which indicates the unmarried women or those women who had not a single parity preceding the survey. Additionally, Family size (classified into less than 5 family members-1, 5 to 6 members-2 and more than 6 members-3), wealth status (poorest-1, poorer-2, middle-3, richer-4 and richest-5) and place of residence (urban-1, rural-2) were some of the important covariates of multimorbidity of women in reproductive age.

### Statistical analysis

First of all, descriptive statistics were performed to give description about the distribution of study participants. After that, percentage distribution was calculated to access the differentials on the prevalence of women’s multimorbidity against different explanatory factors. Association studies were carried out to predict the association and to show the significance level between the dependent and independent variable. The sample weights were employed for estimation of percentage distribution only. Finally, multinominal logistic regression model was conducted to identify the modifiable and non-modifiable risk factors, significantly associated with women multimorbidity. The model was run by considering ‘no morbidity’ as base category for the dependent variable. The results found from multinominal logistic regression were reported as adjusted relative risk ratios (ARRR) in 95% confidence interval and p-values were presented for showing significance level. All statistical outputs were obtained through the Data science software STATA version 15.0 (StataCrop LP, College Station, TX, USA) was used.

## Results

Table 1 is delineated with the distribution of distinctive background characteristics of reproductive sample women (15–49 years) in India. A total of 671,967 women aged between 15 and 49 years were included in this current analysis. Among them, the prevalence of multimorbidity was detected 6.3% (38,535/671,967). One-fourth of the sample women (25.6%) never consumed protein. Only 12% women consumed fruit on daily basis. Half of the sample women consumed green vegetables daily. Among the studied group of women 49% women consumed milk, cud or different dairy products. With this more than 50% of women preferred to have different fried items and 16% women took different aerated drinks. Moreover, 0.4% of the sample women had the habit of tobacco consumption by smoking and 3.4% by chewing, 0.7% women had included the habit of alcohol consumption into their lifestyle. Nearly 7% of sample women were observed obese considering their body mass index.

The proportion of the sample women were noticed maximum (36.4%) in the age cohort of 35–49 years. More than one-third of study participants (34.2%) were recorded as uneducated or having primary education. A substantial proportion of women were from scheduled caste (SC) categories (89.2%), belonging from Hindu communities (82.1%), and living in rural areas (68.5%). Regionally, central region occupied highest percentage share of women (24.9%), followed by eastern (22.3%) and southern region (20.8%).

**Table 1:** Distribution of sample women aged 15–49 years in India, NFHS-5, 2019-21 (*n* = 671,967)

Background Characteristics	Sample (n)	Weighted percentage
**Morbidity types**		
No morbidity	267,124	38.8
Single Morbidity	366,308	54.9
Multimorbidity	38,535	6.3
**Modifiable Factors**		
**Protein Consumption**		
Daily	42,626	6.5
Weekly/occasionally	460,193	67.9
Never	169,148	25.6
**Fruit consumption**		
Daily	80,015	12.3
Weekly/occasionally	582,431	86.1
Never	9521	1.6
**Vegetable consumption**		
Daily	360,884	51.9
Weekly/occasionally	309,343	47.8
Never	1740	0.3
**Milk consumption**		
Daily	309,489	49
Weekly/occasionally	319,356	45.3
Never	43,122	5.7
**Consumption of fried items**		
No	382,876	57.2
Yes	289,091	42.8
**Consumption of aerated drinks**		
No	564,226	84
Yes	107,741	16
**Smoking tobacco**		
No	668,285	99.6
Yes	3682	0.4
**Chewing tobacco**		
No	635,021	96.6
Yes	36,946	3.4
**Alcohol consumption**		
No	659,533	99.3
Yes	12,434	0.7
**Body Mass Index**		
Underweight	120,808	18.4
Normal	404,023	57.8
Overweight	110,887	17.4
Obese	36,249	6.4
**Non-modifiable Factors**		
**Age**		
15–24 years	223,786	33.4
25–34 years	205,089	30.3
35–49 years	243,092	36.4
**Religion**		
Hindu	510,921	82.1
Muslim	82,152	12.9
Christian	47,078	2.3
Others	31,816	2.7
**Caste/tribe**		
Scheduled Caste	545,464	89.2
Scheduled Tribe	94,289	6.1
Others	32,214	4.7
**Geographical Region**		
North	137,530	14.2
Central	158,410	24.9
East	109,297	22.3
North-east	93,336	3.6
West	67,791	14.1
South	105,603	20.8
**Covariates**		
**Educational status**		
No education/primary education	234,301	79.2
Secondary education	343,908	5.4
Higher education	93,758	15.4
**Marital status**		
Unmarried	166,618	23.4
Married	477,415	72.4
Others	27,934	4.2
**Mass media exposure**		
Exposed	512,793	77.5
Not exposed	159,174	22.5
**Parity**		
0	211,781	30.5
1	92,254	14.2
2 or more	367,932	55.4
**Family size**		
< 5 members	266,986	40.2
5–6 members	246,181	35.6
> 6 members	158,800	24.2
**Wealth quintile**		
Poorest	139,063	18.5
Poorer	149,558	20.2
Middle	141,672	20.8
Richer	130,140	20.9
Richest	111,534	19.5
**Place of Residence**		
Urban	162,817	31.5
Rural	509,150	68.5

**Fig. 1:**
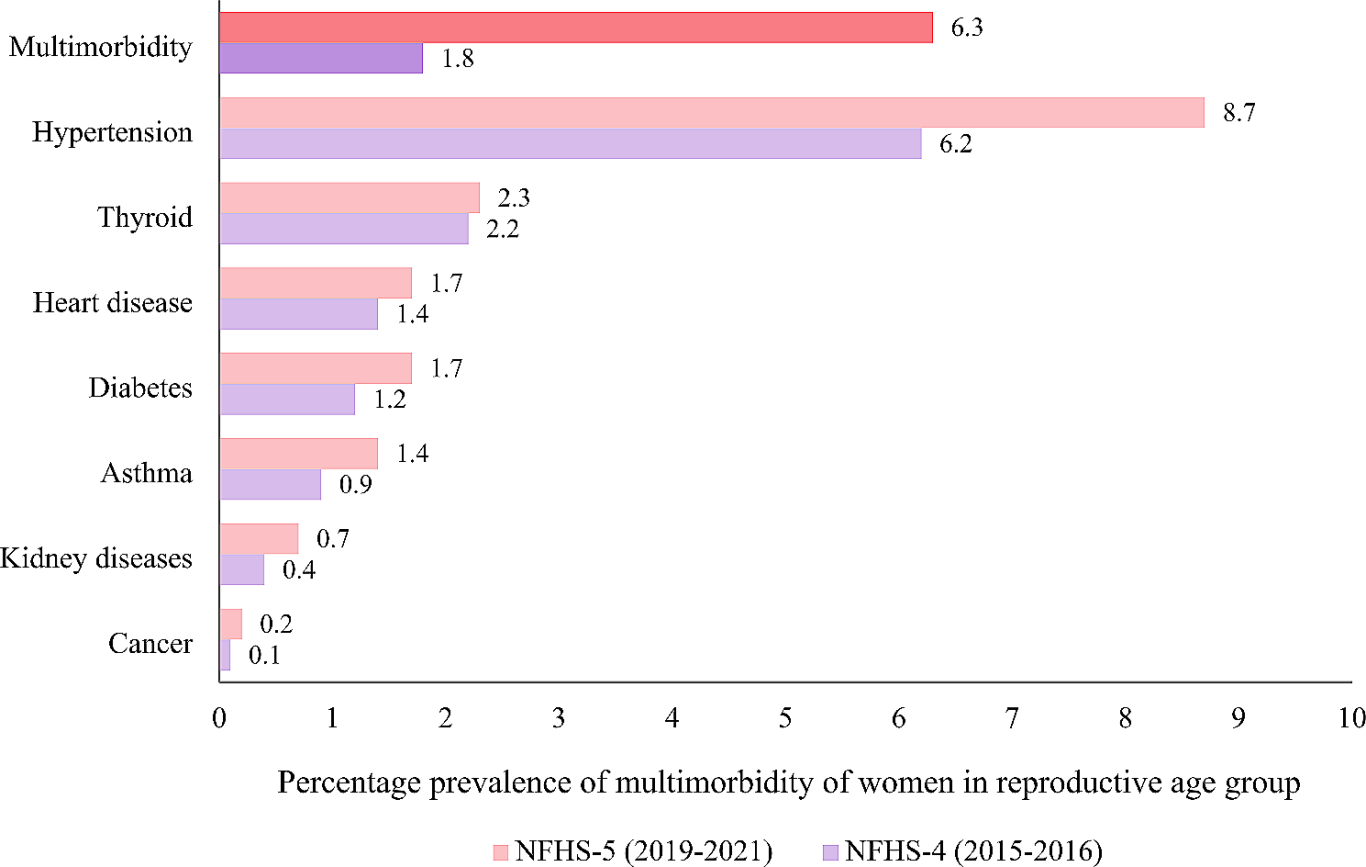
Percentage prevalence of multimorbidity among women aged 15–49 years in India, 2019-21

A comparative scenario regarding the percentage prevalence of different morbidity along with multimorbidity among reproductive age grouped women of two different time period; 2015–2016 and 2019–2021 were illustrated by Fig. 1. This diagram elicited that currently 1.7% of women were suffering with the problem of diabetes whereas the percentage was 1.2 in 2015-16. Among all the morbidities, the prevalence of hypertension was found maximum in both the time period. More than 8 per 100 women of reproductive age had hypertension in recent times. Generally, hypertension is a condition of elevated blood pressure levels which can increase to have the risks of other morbidities also such as heart diseases, stroke, kidney diseases etc. With addition to this, previously only 0.9% women had asthma but now it was shifted to 1.4%. The prevalence of thyroid disorder almost remained same between two comparative time slabs. Currently in NFHS-5, heart diseases, kidney diseases and cancer were found among 1.7%, 0.7%, and 0.2% women respectively which were comparatively less in 2015-16. All the mentioned morbidities among the women of reproductive age showed an increasing proportion in terms of their prevailing percentage. Lastly, the prevalence of multimorbidity among the women aged 15–49 was estimated only 1.8% in 2015-16 which was substantially increased in the future years and the figure became 6.3% in 2019–2021.

Table 2 showed the percentage distribution of women narrates different level of multimorbidity by selected background characteristics. Concerning modifiable factors, the percentage of prevailing multimorbidity was comparatively higher among those women who never consumed protein (64.3%), fruits (83.4%), vegetables (53%) and milk (47.7%). 93.9% of reproductive sample women suffered from multimorbidity who consumed different fried foods and 81.5% sufferer were from those consuming aerated drinks. The incidence of suffering from multimorbidity was higher among those women who had the bad habit of smoking tobacco (99%), chewing tobacco (92.5%) and also alcohol consumption (98.1%). Women Body Mass Index was found to have a strong significant association with the occurrence of multimorbidity. Majority of women suffering from multimorbidity were overweight (25.9%) and obese (49.7%).

Regarding non-modifiable factors, the percentage of women suffering from multimorbidity was gradually increased with increasing age. Women of more than 35 years of age had high prevalence (61.4%) of suffering multimorbidity. Women affiliated to Hindu (71.9%) had higher prevalence of multimorbidity. The multimorbidity prevalence was found more prevalent among those women belonging into others (others-82.9%), apart from scheduled caste and scheduled tribe women. Women in north-east India (26.3%) had high prevalence of multimorbidity than the other regional division in India. Educational status of woman also determines the event of multimorbidity. The uneducated women or the women who have only completed their primary level education (48.2%) had comparatively more risks of suffering from multimorbidity than those having secondary (40.1%) and higher education (11.7%). The prevalence of occurring multimorbidity was relatively higher among the married women (84.1%). The percentage of women aged 15–49 who were exposed to mass media had less chances of being affected by multimorbidity. Mothers having 2 or more children (72.6%) had higher prevalence of multimorbidity. Large households having more than 6 family members (18.6%) had less chances of being affected by multimorbidity than the women attached to small households (47.1%). The multimorbidity prevalence was observed continuously increasing with improving the wealth status of a household. Women from richest (22.6%) families probably found more risks of having multimorbidity as compared to poorest families (14.3%). Rural-urban differentials also made differences in prevailing multimorbidity of women. Rural women (30.4%) had comparatively less risks of having in multimorbid condition than the urban women (69.6%). All the explanatory variables chosen for present analysis are significantly associated with the prevalence of multimorbidity among women in reproductive age group in India.

**Table 2:** Percentage distribution of sample women aged 15–49 years suffering from multimorbidity according to different modifiable and non-modifiable factors in India, 2019-21 (*n* = 671,967)

Background Characteristics	Percentage of Morbidity		*P*-value
	Single Morbidity	Multimorbidity	
**Modifiable Factors**			
**Protein Consumption**			
Daily	7.2	10.5	< 0.001
Weekly/occasionally	25.1	25.3	
Never	67.7	64.3	
**Fruit consumption**			
Daily	1.5	2.1	< 0.001
Weekly/occasionally	11.2	14.5	
Never	87.3	83.4	
**Vegetable consumption**			
Daily	0.2	0.4	< 0.001
Weekly/occasionally	46.1	46.6	
Never	53.7	53	
**Milk consumption**			
Daily	6.7	8	< 0.001
Weekly/occasionally	48.6	44.3	
Never	44.7	47.7	
**Consumption of fried items**			
No	4.6	6.1	< 0.001
Yes	95.4	93.9	
**Consumption of aerated drinks**			
No	16.3	18.5	< 0.001
Yes	83.7	81.5	
**Smoking tobacco**			
No	0.5	1	< 0.001
Yes	99.5	99	
**Chewing tobacco**			
No	5.5	7.5	< 0.001
Yes	94.5	92.5	
**Alcohol consumption**			
No	1.9	1.9	< 0.001
Yes	98.1	98.1	
**Body Mass Index**			
Underweight	4.6	14	< 0.001
Normal	20.2	10.4	
Overweight	14.7	25.9	
Obese	60.4	49.7	
**Non-modifiable Factors**			
**Age**			
15–24 years	30.3	12.5	< 0.001
25–34 years	34.8	26.1	
35–49 years	34.9	61.4	
**Religion**			
Hindu	77.7	71.9	< 0.001
Muslim	5.5	5.9	
Christian	12.2	15.7	
Others	4.6	6.5	
**Caste/tribe**			
Scheduled Caste	13.6	9.7	< 0.001
Scheduled Tribe	5	7.4	
Others	81.5	82.9	
**Geographical Region**			
North	12.5	13.5	< 0.001
Central	18.7	18.1	
East	23.1	14.7	
North-east	20.4	26.3	
West	10.7	8.2	
South	14.6	19.2	
**Covariates**			
**Educational status**			
No education/Primary education	51.2	48.2	< 0.001
Secondary education	36	40.1	
Higher education	12.8	11.7	
**Marital status**			
Unmarried	25.3	8.9	< 0.001
Married	70.6	84.1	
Others	4.1	7	
**Mass media exposure**			
Exposed	25.2	18.8	< 0.001
Not exposed	74.8	81.2	
**Parity**			
0	13.7	13.7	< 0.001
1	31.8	13.7	
2 or more	54.6	72.6	
**Family size**			
< 5 members	38.8	47.1	< 0.001
5–6 members	37.1	34.3	
> 6 members	24.1	18.6	
**Wealth quintile**			
Poorest	23	14.3	< 0.001
Poorer	22.8	18.9	
Middle	20.8	21.2	
Richer	18.4	23.1	
Richest	15.1	22.6	
**Place of Residence**			
Urban	77.6	69.6	< 0.001
Rural	22.4	30.4	

Table 3 denoted results of multinominal logistic regression analysis to identify the risk factors associated with multimorbid conditions among the women of reproductive aged between 15 and 49 years. Here, no morbidity condition has been taken as a base outcome category. The first column of the table showed the results for ‘single morbidity’ compared to ‘no morbidity’ whereas the results of ‘multimorbidity’ compared to ‘no morbidity’ was represented by the second column of the table. With no morbidity as the base outcome, the results revealed that women who never consumed protein, fruits, vegetable and milk had an increased likelihood of suffering from single morbidity than those eating these foods daily. Similarly, by considering no morbidity as the base category, the women who consumed protein rich food once in a week or occasionally (ARRR: 1.860, 95% CI: 1.827–1.894) were more likely to be affected by multimorbidity than those consuming daily and the likelihood was observed much more than daily/occasionally among those women never consumed protein (ARRR: 1.925, 95% CI: 1.882–1.969). The study further observed that weekly or occasional consumption of different food items required for healthy life had comparatively higher likelihood (fruits-24%, vegetables-6% and milk-12%) of prevailing multimorbidity compared to those consuming these foods daily. The likelihood of suffering multimorbidity was 38% higher among the women who never consumed fruits and similarly with this, the women who never consumed vegetables and milks were 63% and 61% more likely to develop multimorbidity. The women who ate different fried items were more susceptible to be affected by single morbidity (ARRR: 1.093, 95% CI: 1.066–1.122) and multimorbidity (ARRR: 1.292, 95% CI: 1.230–1.357) than those who did not. Similarly, consumption of different aerated drinks also has a significant association with morbidity prevalence. Women who consumed tobacco by smoking or chewing were 51% and 39% more likely to be associated with women’s multimorbidity. Likewise, 28% more likelihood was prevalent in case of women consuming alcohol. However, the probability of suffering from single and multimorbidity is increased gradually with increasing BMI. The overweight (ARRR: 1.206; 95% CI: 1.156–1.257) and obese women (ARRR: 2.052; 95% CI: 1.954–2.154) were more likely to suffer from multimorbidity than the thin or lean women. Not only that, one important thing was observed that women having normal BMI had lesser chances of affecting by whatever single or multimorbidity than the thin, overweight and obese women.

The results further revealed that different non-modifiable factors also have a significant influence on the prevalence of women’s multimorbidity. Women’s age had found a strong and statistically significant association with women’s multimorbidity. Women with continuously increasing ages became more susceptible to suffer from single and multimorbidity. Women falling from the age group of 35–49 years were 2 times and almost 3 times more likely to have single morbidity and multimorbidity than the reference category which was 15–24 years. The Muslim women were less likely to develop single morbidity than the Hindu communities. Similar results were found in case of affecting by multimorbidity.The women belonging from other categories were more susceptible to be affected by single (ARRR: 1.201, 95% CI: 1.170–1.233) and multimorbidity (ARRR: 1.497, 95% CI: 1.427–1.520) than the SC and ST women. The results of this analysis further revealed that the prevalence of single morbidity and multimorbidity of women aged 15–49 was comparably higher among the women hailed from eastern and north-eastern part of India.

**Table 3:** Multinominal Logistic Regression Model estimating modifiable and non-modifiable factors associated with multimorbidity among women (15–49 years) in India, NFHS-5, 2019-21

Predictor Variables	Base outcome (No morbidity)	
	Single morbidity	Multimorbidity
	ARRR (95% CI)	ARRR (95% CI)
**Modifiable Factors**		
**Protein Consumption**		
Daily	Ref.	Ref.
Weekly/occasionally	1.015* (0.995–1.036)	1.860* (1.827–1.894)
Never	1.023* (0.999–1.047)	1.925* (1.882–1.969)
**Fruit consumption**		
Daily	Ref.	Ref.
Weekly/occasionally	1.002* (0.969–1.003)	1.236* (1.136–1.289)
Never	1.030* (0.983–1.080)	1.381* (1.266–1.507)
**Vegetable consumption**		
Daily	Ref.	Ref.
Weekly/occasionally	1.042* (1.031–1.053)	1.062* (1.037–1.087)
Never	1.043* (0.943–1.153)	1.638* (1.361–1.972)
**Milk consumption**		
Daily	Ref.	Ref.
Weekly/occasionally	1.056* (1.044–1.068)	1.122* (1.095–1.151)
Never	1.122* (1.097–1.148)	1.613* (1.541–1.687)
**Consumption of fried items**		
No	Ref.	Ref.
Yes	1.093 (1.066–1.122)	1.292* (1.230–1.357)
**Consumption of aerated drinks**		
No	Ref.	Ref.
Yes	1.072 (1.012–1.099)	1.108* (1.075–1.142)
**Smoking tobacco**		
No	Ref.	Ref.
Yes	1.255* (1.168–1.342)	1.514* (1.350–1.699)
**Chewing tobacco**		
No	Ref.	Ref.
Yes	1.092 (1.066–1.118)	1.393* (1.330–1.458)
**Alcohol consumption**		
No	Ref.	Ref.
Yes	1.084* (0.997–1.179)	1.284* (1.197–1.379)
**Body Mass Index**		
Underweight	Ref.	Ref.
Normal	0.822* (0.811–0.834)	0.915* (0.882–0.950)
Overweight	1.311* (1.259–1.386)	1.206* (1.156–1.257)
Obese	1.778* (1.757–1.799)	2.052* (1.954–2.154)
**Non-modifiable Factors**		
**Age**		
15–24 years	Ref.	Ref.
25–34 years	1.591* (1.523–1.663)	1.527* (1.459–1.597)
35–49 years	2.138* (2.107–2.275)	2.858* (2.726–2.995)
**Religion**		
Hindu	Ref.	Ref.
Muslim	0.622* (0.602–0.643)	0.601* (0.561–0.643)
Christian	1.021* (0.992–1.051)	1.078 (1.019–1.140)
Others	1.043* (1.017–1.069)	0.822 (0.783–0.863)
**Caste/tribe**		
Scheduled Caste	Ref.	Ref.
Scheduled Tribe	1.067 (1.048–1.087)	0.767 (0.733–0.802)
Others	1.201* (1.170–1.233)	1.497* (1.427–1.520)
**Geographical Region**		
North	Ref.	Ref.
Central	0.975 (0.954–0.996)	0.665 (0.633–0.699)
East	1.569* (1.534–1.604)	1.671* (1.595–1.751)
North-east	1.237* (1.210–1.266)	1.247* (1.189–1.309)
West	1.262 (1.232–1.294)	0.778 (0.737–0.822)
South	1.067 (1.042–1.092)	0.962* (0.917–1.010)
**Covariates**		
**Educational status**		
No education/primary education	Ref.	Ref.
Secondary education	0.980 (0.965–0.995)	1.080 (1.046–1.114)
Higher education	0.878 (0.860–0.897)	0.904* (0.863–0.947)
**Marital status**		
Unmarried	Ref.	Ref.
Married	1.253* (1.119–1.321)	1.362* (1.281–1.448)
Others	1.561* (1.476–1.587)	1.752* (1.626–1.888)
**Mass media exposure**		
Exposed	Ref.	Ref.
Not exposed	1.132 (1.113–1.276)	1.294 (1.189–1.342)
**Parity**		
0	Ref.	Ref.
1	1.149* (1.122–1.176)	1.203* (1.137–1.272)
2 or more	1.188* (1.162–1.214)	1.294* (1.228–1.364)
**Family size**		
< 5 members	Ref.	Ref.
5–6 members	1.024 (1.012–1.036)	0.914* (0.891–0.937)
> 6 members	1.019 (1.005–1.033)	0.842* (0.816–0.868)
**Wealth quintile**		
Poorest	Ref.	Ref.
Poorer	1.089* (1.057–1.099))	1.081* (1.040–1.125)
Middle	1.123* (1.089–1.211)	1.170* (1.123–1.220)
Richer	1.231* (1.221.142)	1.285* (1.229–1.343)
Richest	1.298* (1.276–1.354)	1.309* (1.244–1.377)
**Place of Residence**		
Urban	Ref.	Ref.
Rural	1.164 (1.045–1.234)	1.005 (0.973–1.028)

ARRR = Adjusted Relative Risk Ratios; CI = Confidence Interval; Ref.-Reference Category;

*Significant at *p* < 0.05

## Discussion

On the basis of nationally representative NFHS-5 data, the study made an effort to extensively explore some key modifiable and non-modifiable risk factors of multimorbidity conditions develop among reproductive age-grouped women in Indian context. Major findings of this study highlighted the fact that 6.3 per 100 women in reproductive age group suffered from two or more morbidities. Findings further suggested that different modifiable factors comprising protein intake, fruits, vegetables and milk consumption, consumption of fried foods and aerated drinks, tobacco consumption (smoking & chewing), alcohol consumption and BMI and while different non-modifiable factors comprising age of women, their religious background, caste/tribe and geographical region have statistically significant association with the prevalence of multimorbidity of women.

According to the study’s findings, greater prevalence of women’s multimorbidity was noticed among those women, never consuming protein, fruits, vegetables and milk [22] & [37]. Evidence suggested that healthy diet rich in nutritious foods like fruits and vegetables can control elevated blood pressure level, lower the chance of heart disease and stroke, can prevent some sorts of cancer and do have a great positive effect on diabetic patients which in terms helps to reduce the risks of prevailing multimorbidity [38] & [39]. This could be explained as fruits and vegetables contain vitamins, minerals, antioxidants and phyto chemicals which may help to protect and prevent from various diseases. The study further revealed that unhealthy diet like consumption of fried foods raised the chances of developing multimorbidity among the sample women. A previous review studies based on current evidence reflected that consumption of fried foods worsens cardiovascular health and constitutes a big reason of heart failure. Not only that, hypertension, diabetes and obesity can be resulted from fried food consumption which can be regulated for evolving the other different morbidities also [40]. Generally fried foods are contained high in fat (saturated fat and Tran’s fat), calories and cholesterol which are very much harmful for every human body, may lead to develop several life-threatening diseases at a time. Tobacco consumption and alcohol consumption have a large effect on the prevalence of multimorbidity among reproductive age grouped women that is also incorporated with some other earlier studies [22] & [41]. As evidence suggested, smoking causes several diseases such as cancer, lung disease, heart disease, stroke, diabetes, severe asthma which in turn can lead to develop multimorbidity among the smokers [42, 43] & [44]. This could be demonstrated by the fact that smoking habits can harm inherent immune cells and suppress the creation of immunological molecules, accelerating to the quick circulation and deep-rooted colonization of pathogens [45] & [46]. Likewise, harmful use of alcohol can cause serious health effects among the women. According to World Health Organization, drinking alcohol is severely associated with major non-communicable diseases such as liver cirrhosis, heart diseases and certain cancers. Earlier studies highlighted a correlation between excessive alcohol consumption and dysfunction of immune system [47]. Additionally, past studies examined that women are more vulnerable to different alcohol-related health hazards than men. Drinking same quantity of alcohol, women have relatively higher blood alcohol levels and the immediate effects of drinking alcohol generally ensue more rapidly and last for longer period in women than men, make them vulnerable to several long term negative health consequences [48].This study also examined a substantial influence of women’s Body Mass Index on developing multimorbidity among them. The obese women were found 2 times more likely to be affecting by multimorbidity than the slim women, consistent with several other previous studies [49] & [50]. Obesity increases the chances of prevailing manifold debilitating diseases comprising diabetes, heart diseases, high blood pressure, high cholesterol, liver disease and some cancers, was caused due to the mechanical stress of bearing extra body fats, or due to the multiplex changes of hormone and metabolism.

Predominance of multimorbidity among women is found in the subsequent phases of their reproductive span. This finding has been validated by some other previous studies conducted in India [51] & [52].When women reach at the later stage of reproductive years, it starts to deplete the level of estrogen from the body. This depletion of estrogen levels can cause alteration and modification on biological well-bring and can also amplify the chances of morbidity appearance. Due to the factors of parity and experiencing menopause before reaching the age of natural menopause, the probability of occurring multimorbidity accelerates with accelerating reproductive ages [53] & [54]. Apart from that, with increasing age immune system of every human beings weakens, makes vulnerable to different diseases. Our study further examined that scheduled tribe women are less affected by multimorbidity compared to other categories [55]. This could be described as most of the scheduled tribe women are engaged into primary and secondary economic activities where more than enough physical activities are done and they consume fresh food mainly green fresh vegetables and fruits collected from the field. Another finding revealed in the current study that the burden of multimorbidity was observed higher among the women from eastern and north-eastern India compared to other parts of India. The people of eastern and north-eastern India generally face various challenges such as lack of infrastructure, poor social status, backward economy and demographic issues. At the same time, they have poor literacy rates; most of the women are confined indoors, poor nutrition, lack of access to medical facilities which may make them prone to develop multimorbidity in their reproductive period.

The current study faced a few limitations. This study included only seven chronic illnesses for multimorbidity analysis. NFHS-5 did not provide information about many other chronic diseases (arthritis, stroke, paralysis, back pain etc.) which also would impact the health conditions of women aged 15–49 years, resulting into under-representing the issue. Lack of information on several diseases is the major drawback of this study. However, physical activity is an important determining modifiable factors of multimorbidity, but NFHS-5 did not provide any information related to physical activity, could not be taken into the study.

## Conclusion

The study findings explore and indicate the different modifiable and non-modifiable factors responsible for multimorbidity in the context of women of reproductive age group (15–49 years) in India. Currently, India is facing a greater prevalence of multimorbidity among the women of reproductive ages that may rise gradually over the future decades. As reproductive span is the foundation of every woman’s health in later life, it becomes necessary to provide required age-specific health care facilities to those women affected by multiple morbidities. At the same time, to reduce the risk of developing multimorbidity, targeted interventions in the form of educating every woman concerning the importance of having minimum health-related knowledge, maintaining healthy lifestyle, weight management and having proper and balanced diet becomes crucial. Besides, our study suggests that with focusing on sexual and reproductive health aspects of women aged 15–49 years, Indian Government should also emphasis on adopting various national programmes incorporating management of chronic illnesses of women in reproductive agesto prevent and minimize this multimorbidity burden in our country.

## Data Availability

No datasets were generated or analysed during the current study.
